# Parental Knowledge and Attitude Toward Childhood Hearing Loss and Hearing Services in Tabuk, Saudi Arabia: A Cross-Sectional Study

**DOI:** 10.7759/cureus.96691

**Published:** 2025-11-12

**Authors:** Saad Hamdi Eid Alenzi, Nourah Eid A Alatwi, Abdulrahman Abdullah S Alelyani, Hatoun Alali, Farah Abdulrahman S Aljohani, Hassan Ayman H Aljohani, Abdullah Nasser A Alayda, Khalid Bakhet B Aljohani, Lama Mousa A Alenazi, Abdullah Ahmed M Alhejaily, Rawan Saud Alshahrani, Faisal Abdallah Alhejji, Ruba Mahmoud A Almuallim, Rahaf Hamdan A Albalawi, Deema F Alharbi

**Affiliations:** 1 Department of Otorhinolaryngology-Head and Neck Surgery, King Fahad Specialist Hospital, Tabuk, SAU; 2 Department of Otorhinolaryngology-Head and Neck Surgery, College of Medicine, University of Tabuk, Tabuk, SAU

**Keywords:** attitude, childhood, hearing loss, hearing services, knowledge, saudi arabia

## Abstract

Background

Childhood hearing loss (HL) can impede development and learning and requires early intervention. Community support, particularly parental awareness, is essential for effective audiology services. Assessing this awareness is crucial for designing appropriate hearing programs for children. Therefore, this study aimed to assess the knowledge and attitudes regarding childhood HL and hearing services among the adult population in Tabuk, Saudi Arabia.

Methods

This cross-sectional study involved 401 adult male and female participants aged 20 years and older, residing in Tabuk, Saudi Arabia. Data were gathered through a self-administered online questionnaire comprising demographic details and 26 items evaluating parental knowledge and attitudes toward HL. Total scores for knowledge and attitude were calculated, and the relationships between sociodemographic factors and levels of knowledge and attitudes toward HL were analyzed.

Results

Among all participants, 241 (60.1%) demonstrated a good level of knowledge, while 293 (73.1%) exhibited positive attitudes toward audiology services. Significant associations were observed between knowledge level and both age (p = 0.003) and sex (p = 0.008). Female participants accounted for 55.6% of those with positive attitudes, whereas male participants comprised 63.0% of those with negative attitudes, with a statistically significant difference (p < 0.001). Furthermore, widowed participants were significantly more likely to exhibit negative attitudes (10.2%) compared to only 2.4% among those with positive attitudes.

Conclusion

This study explored gaps in parental knowledge and awareness of childhood HL in Tabuk, Saudi Arabia, particularly regarding specific risk factors. While overall attitudes toward hearing services were generally positive, targeted educational efforts are needed to improve awareness and foster more favorable attitudes among men and widowed individuals.

## Introduction

Childhood hearing loss (HL) is a major public health concern in developing countries, as it negatively impacts the psychological and social well-being of both children and their families. Undiagnosed HL can lead to delays in language development, communication difficulties, reduced academic performance, limited social interaction, and emotional distress. Families of affected children may also experience increased stress, financial burden, and social isolation, particularly in settings where access to hearing healthcare and support services is limited [1].

HL is among the most prevalent disabilities globally [2]. It is observed in about 1 to 3 out of every 1,000 newborns [3]. Among children, the prevalence of HL ranges from 7.7% to 15.5%, varying based on the diagnostic criteria used [4]. Additionally, permanent congenital HL in children is reported in about 1.2 to 1.7 cases per 1,000 live births [5,6].

HL may be caused by a wide variety of congenital and acquired factors [7]. Certain risk factors, such as neurodegenerative disorders and congenital infections and postnatal hypoxia, place a child at significantly greater risk of congenital hearing impairment or developing permanent HL [8,9].

Newborn hearing screening programs are designed to test all infants within the first month of life. However, these programs may not detect cases of progressive HL that develop later. As a result, implementing regular hearing screenings for school-aged children is essential to identify those who were either missed at birth or who developed hearing impairments over time. In Saudi Arabia, a National Newborn Hearing Screening Program has been established to ensure early detection and intervention for infants with hearing impairment; however, extending periodic screening to older children would further enhance early identification of progressive or late-onset cases and support timely management [10].

Early detection, together with appropriate intervention, is critical to improve speech, language, and cognitive development. However, it is often a challenge due to a lack of awareness and low rates of screening at birth [11,12]. In addition, parental reluctance to accept that their child may have a hearing impairment can delay diagnosis and hinder progress towards hearing aid treatments. Previous studies have indicated that parents generally support the importance of early detection and intervention for hearing impairments [13,14].

Community knowledge and attitudes play a pivotal role in the uptake of hearing screening programs and support services. Understanding public perception can help inform health education strategies and policy development aimed at improving childhood hearing health and quality of life [15]. Therefore, this study aimed to assess the knowledge and attitudes regarding childhood HL and hearing services among the adult population in Tabuk, Saudi Arabia.

## Materials and methods

Study design, settings, and duration

This community-based cross-sectional survey study was carried out in Tabuk, Saudi Arabia, throughout the period from January 2025 to June 2025.

Ethical considerations

The study was conducted following the ethical principles outlined in the Declaration of Helsinki. Ethical approval was obtained from the institutional Research Ethics Committee of the University of Tabuk (Approval number: UT-476-281-2024). Before starting the questionnaire, each participant viewed an information sheet describing the study aims, voluntary participation, anonymity, and data confidentiality. Participants gave consent by selecting the ‘I agree’ option and proceeding to complete the online survey; only those who provided consent could access the questionnaire. To ensure anonymity, data were analyzed using coded identifiers assigned to each participant.

Sample size and sampling method

The sample size was estimated with an online sample size calculator (Raosoft, http://www.raosoft.com/samplesize.html) using a margin of error of 5%, a confidence interval of 95%, response distribution of 50%, and depending on an average population size of nearly 560,000 adults in Tabuk, Saudi Arabia, according to the Saudi Census 2023 [16]. The minimum required sample size was 384. However, a total of 401 eligible participants were included in the analysis. A non-probability convenience sampling technique was applied to recruit the participants.

Eligibility criteria

The study included adult male and female individuals aged 20 years and above, residing in Tabuk, Saudi Arabia. Subjects younger than 20 years, those who refused to participate, and questionnaires with incomplete responses were excluded from the analysis.

Data collection

Data were collected using a structured online survey developed via the “Google Forms” platform. This was a community-based cross-sectional survey conducted among adult residents of Tabuk, Saudi Arabia. Invitations for participation were sent through various social media channels, including Facebook, Instagram, and WhatsApp groups. The survey was administered in Arabic and designed based on prior research to evaluate participants’ knowledge and attitudes [17-21]. The questionnaire consisted of three sections. The first section gathered sociodemographic details such as age, sex, nationality, educational level, marital status, number of children, monthly income (in Saudi Riyals), and whether the respondent had a medical background. The second section assessed knowledge through 21 questions addressing risk factors, otitis media, HL detection, and intervention. The questionnaire was translated into Arabic using a forward-backward approach. Two bilingual experts prepared independent forward translations, which were reconciled and back-translated into English by two other bilingual translators blinded to the original. A committee reviewed all versions, resolved discrepancies, and produced a pre-final Arabic version, which was piloted to assess clarity, ease of use, and completion time.

Each question offered three response options: "yes," "no," and "don’t know." A score of 1 was assigned to "yes" responses, while "no" and "don’t know" received a score of 0. The cumulative knowledge score was based on adding the 21 items, with scores categorized as “poor knowledge” (≤11 points) or “good knowledge” (≥12 points). The third section comprised five questions evaluating attitudes toward seeking audiological healthcare services. Responses were scored with 1 point for "yes" and 0 for either "no" or "unsure". Attitude scores were classified as “negative” (0-3 points) or “positive” (4-5 points). Don’t know” and "unsure" responses were scored 0 because both reflect the absence of correct knowledge or an affirmative attitude, and this ensures that only definite, evidence-based responses contributed to higher scores. The cut-off points for knowledge and attitude scores depended on using a 50% threshold of the maximum possible score, which is a commonly applied criterion in such studies when validated benchmarks are not available.

Statistical analysis

Data were entered and analyzed using IBM SPSS Statistics for Windows, Version 27 (Released 2020; IBM Corp., Armonk, New York, United States). Questionnaires with incomplete responses were excluded from the analysis (complete-case approach); no imputation of missing values was performed. Descriptive statistics were applied to summarize the data, with categorical variables presented as frequencies and percentages. Associations between sociodemographic variables and participants’ levels of knowledge and attitudes were assessed using the chi-square test, Fisher’s exact test, or the Fisher-Freeman-Halton Exact test, as appropriate based on data distribution and expected cell counts. When the Fisher-Freeman-Halton Exact test indicated statistical significance, post hoc pairwise comparisons were conducted using the Bonferroni-adjusted Z-test to identify specific categories contributing to the observed differences. A p-value of <0.05 was considered statistically significant.

## Results

The sociodemographic characteristics of the 401 study participants indicate a predominantly young to middle-aged group, with 217 individuals (54.1%) aged 20-39 years and 162 (40.4%) aged 40-59 years. The gender distribution was nearly equal, with 203 female participants (50.6%) and 198 male participants (49.4%). A large majority were Saudi nationals (362, 90.3%), and most were married (348, 86.8%). Regarding family size, 240 (59.9%) participants had three or more children. Educational level was generally high, with 301 (75.1%) participants holding a university degree or higher. In terms of monthly income, 166 (41.4%) respondents reported earning more than 10,000 Saudi Riyals, while 122 (30.4%) earned between 5,000 and 10,000, and 113 (28.2%) earned less than 5,000. Additionally, 149 (37.2%) members reported having a medical background, while 252 (62.8%) did not (Table 1).

**Table 1:** Sociodemographic characteristics of the study participants (N=401) N: number

		N=401	%
Age, year	20-39	217	54.1%
	40-59	162	40.4%
	≥ 60	22	5.5%
Sex	Female	203	50.6%
	Male	198	49.4%
Nationality	Saudi	362	90.3%
	Non-Saudi	39	9.7%
Marital status	Married	348	86.8%
	Divorced	35	8.7%
	Widowed	18	4.5%
Number of children	1-2	161	40.1%
	≥3	240	59.9%
Education level	Secondary school or below	100	24.9%
	University or above	301	75.1%
Monthly income, Saudi Riyal	< 5000	113	28.2%
	5000 -10000	122	30.4%
	> 10000	166	41.4%
Medical background	Yes	149	37.2%
	No	252	62.8%

Table 2 shows participants' awareness and attitudes toward childhood HL and related risk factors. The findings reveal that a high proportion of participants were aware that babies can be born with HL (85.3%) and that factors such as head trauma (76.3%), ear discharge or otitis media (73.6%), family history (70.8%), consanguineous marriage (65.1%), and noise exposure (66.1%) can contribute to HL. Additionally, 82.3% believed that children with HL can attend schools, and 71.1% recognized speech or language problems as potential signs of HL. There was a fair level of knowledge about preventive and diagnostic measures, such as the role of breastfeeding for six months (61.6%), as well as routine childhood immunization in reducing OM (57.1%), identification of HL soon after birth (55.9%), and the availability of treatment (59.4%). However, awareness was lower regarding less obvious causes such as chemotherapy (38.2%), radiotherapy (41.9%), jaundice (21.7%), and low birth weight (28.4%), with over half of participants expressing uncertainty about these items. Regarding attitudes toward hearing services, most parents expressed a desire to receive more information about childhood HL and services (88.5%), were in favor of having their child tested at school (87.3%), supported testing their baby soon after birth (84.0%), and were willing to let their child use hearing aids (74.6%). However, acceptance was relatively lower for more invasive measures, such as ear surgery (67.3%).

**Table 2:** Responses of the study participants to the knowledge and attitude statements (N=401) HL: hearing loss; OM: otitis media

Knowledge Items	No		Don’t know/Unsure		Yes	
	N	%	N	%	N	%
1. Babies can be born with HL	8	2.0%	51	12.7%	342	85.3%
2. Maternal infection can cause HL	47	11.7%	146	36.4%	208	51.9%
3. Neonatal infection can cause HL	57	14.2%	154	38.4%	190	47.4%
4. CNS infection can cause HL	34	8.5%	133	33.2%	234	58.4%
5. Drugs/medications can cause HL	47	11.7%	172	42.9%	182	45.4%
6. Radiotherapy can cause HL	34	8.5%	199	49.6%	168	41.9%
7. Chemotherapy can cause HL	44	11.0%	204	50.9%	153	38.2%
8. Jaundice can cause HL	76	19.0%	238	59.4%	87	21.7%
9. Family history can be associated with HL	23	5.7%	94	23.4%	284	70.8%
10. Consanguineous marriage can be associated with HL	35	8.7%	105	26.2%	261	65.1%
11. Low birth weight	81	20.2%	206	51.4%	114	28.4%
12. Noise exposure can cause HL	49	12.2%	87	21.7%	265	66.1%
13. Head trauma can cause HL	14	3.5%	81	20.2%	306	76.3%
14. OM and ear discharge can cause HL	19	4.7%	87	21.7%	295	73.6%
15. Breast-feeding for first 6 months can reduce/prevent OM	25	6.2%	129	32.2%	247	61.6%
16. Smoking can predispose to OM	58	14.5%	192	47.9%	151	37.7%
17. Routine childhood immunizations can reduce OM	40	10.0%	132	32.9%	229	57.1%
18. HL can be identified soon after birth	66	16.5%	111	27.7%	224	55.9%
19. Speech/language problems can be a sign of HL	33	8.2%	83	20.7%	285	71.1%
20. There is available treatment for HL	21	5.2%	142	35.4%	238	59.4%
21. Children with HL can attend schools	9	2.2%	62	15.5%	330	82.3%
Attitude items						
1. I would like my baby tested soon after birth	14	3.5%	50	12.5%	337	84.0%
2. I would like my child tested at school	21	5.2%	30	7.5%	350	87.3%
3. I would let my child use hearing aids	19	4.7%	83	20.7%	299	74.6%
4. I would accept ear surgery for my child	29	7.2%	102	25.4%	270	67.3%
5. I would like more information	13	3.2%	33	8.2%	355	88.5%

Table 3 shows that the prevalence of good knowledge level was 60.1%, while poor knowledge constituted 39.9%. There were some significant differences in the distribution of sociodemographic characteristics between the poor and good knowledge groups. The age showed a significant association with knowledge level (p=0.003), obviously in the older age group, with higher poor than good knowledge among those aged 60 or older (10% vs 2.5%, respectively). Regarding sex, females comprised 42.5% of the poor knowledge group but 56.0% of the good knowledge group, while males were 57.5% in the poor group versus 44.0% in the good knowledge group, with a significant difference (p=0.008). Nationality, marital status, number of children, education level, and monthly income showed no significant differences between knowledge groups (All p>0.05). Lastly, for medical background, 29.4% of those with poor knowledge reported having a medical background, compared to 42.3% in the good knowledge group, with a statistically significant difference (p=0.009).

**Table 3:** Associations between knowledge levels and sociodemographic characteristics of the study participants (N=401) *Significant at p<0.05.

		Knowledge level				Overall knowledge N=401 (100%)		Test statistic X^2^	p-value
		Poor N=160 (39.9%)		Good N=241 (60.1%)					
Age, year	20-39	88	55.0%	129	53.5%	217	54.1%	11.846	0.003*
	40-59	56	35.0%	106	44.0%	162	40.4%		
	≥ 60	16	10.0%	6	2.5%	22	5.5%		
Sex	Female	68	42.5%	135	56.0%	203	50.6%	7.028	0.008*
	Male	92	57.5%	106	44.0%	198	49.4%		
Nationality	Saudi	147	91.9%	215	89.2%	362	90.3%	0.777	0.378
	Non-Saudi	13	8.1%	26	10.8%	39	9.7%		
Marital status	Married	136	85.0%	212	88.0%	348	86.8%	1.937	0.380
	Divorced	14	8.8%	21	8.7%	35	8.7%		
	Widowed	10	6.3%	8	3.3%	18	4.5%		
Number of children	1-2	69	43.1%	92	38.2%	161	40.1%	0.981	0.322
	≥3	91	56.9%	149	61.8%	240	59.9%		
Education level	Secondary school or below	37	23.1%	63	26.1%	100	24.9%	0.467	0.494
	University or above	123	76.9%	178	73.9%	301	75.1%		
Monthly income, Saudi Riyal	< 5000	42	26.3%	71	29.5%	113	28.2%	0.490	0.783
	5000-10000	50	31.3%	72	29.9%	122	30.4%		
	> 10000	68	42.5%	98	40.7%	166	41.4%		
Medical background	Yes	47	29.4%	102	42.3%	149	37.2%	6.905	0.009*
	No	113	70.6%	139	57.7%	252	62.8%		

Table 4 demonstrates that a substantial proportion of participants (73.1%) exhibited a positive attitude. Female participants represented 55.6% of the positive attitude group compared to 37.0% in the negative attitude group, while male participants constituted 63.0% of the negative group versus 44.4% of the positive group, with a significant difference (p<0.001). Additionally, widowed subjects were significantly more likely to have a negative attitude (10.2%) compared to only 2.4% in the positive attitude group (p = 0.003). No other significant associations were observed between participants’ attitude and their age, nationality, number of children, educational level, income, or medical background (All p > 0.05).

**Table 4:** Associations between attitudes of the study participants and sociodemographic characteristics (N=401) *Significant at p<0.05.

		Attitude level				Overall attitude N=401 (100%)		Test statistic X^2^	p-value
		Negative N=108 (26.9%)		Positive N=293 (73.1%)					
Age, year	20-39	53	49.1%	164	56.0%	217	54.1%	1.600	0.449
	40-59	49	45.4%	113	38.6%	162	40.4%		
	≥ 60	6	5.6%	16	5.5%	22	5.5%		
Sex	Female	40	37.0%	163	55.6%	203	50.6%	10.915	<0.001*
	Male	68	63.0%	130	44.4%	198	49.4%		
Nationality	Saudi	94	87.0%	268	91.5%	362	90.3%	1.764	0.184
	Non-Saudi	14	13.0%	25	8.5%	39	9.7%		
Marital status	Married	90	83.3%	258	88.1%	348	86.8%	11.742	0.003*
	Divorced	7	6.5%	28	9.6%	35	8.7%		
	Widowed	11	10.2%	7	2.4%	18	4.5%		
Number of children	1-2	43	39.8%	118	40.3%	161	40.1%	0.007	0.934
	≥3	65	60.2%	175	59.7%	240	59.9%		
Education level	Secondary school or below	22	20.4%	78	26.6%	100	24.9%	1.674	0.199
	University or above	86	79.6%	215	73.4%	301	75.1%		
Monthly income, Saudi Riyal	< 5000	26	24.1%	87	29.7%	113	28.2%	3.609	0.165
	5000-10000	29	26.9%	93	31.7%	122	30.4%		
	> 10000	53	49.1%	113	38.6%	166	41.4%		
Medical background	Yes	32	29.6%	117	39.9%	149	37.2%	3.587	0.058
	No	76	70.4%	176	60.1%	252	62.8%		

## Discussion

HL can pose a major obstacle to both education and social inclusion. Without timely support, children with HL may experience delays in language development, reduced academic performance, and difficulty forming peer relationships, which can lead to long-term educational and psychosocial challenges. Early identification and intervention are essential to improve outcomes. By promoting awareness, ensuring access to hearing services, and promoting supportive environments, children with HL can actively engage in both learning and community.

In this study, the parents residing in Tabuk City, Saudi Arabia, exhibited a generally moderate level of knowledge regarding childhood HL, with 60.1% demonstrating a good knowledge level. Most participants had a considerable awareness of common risk factors for childhood HL, including congenital causes (85.3%), head trauma (76.3%), otitis media (73.6%), family history (70.8%), consanguinity (65.1%), and noise exposure (66.1%). However, there were notable knowledge gaps about the less obvious risk factors like chemotherapy (38.2%), radiotherapy (41.9%), jaundice (21.7%), low birth weight (28.4%), neonatal infection (47.4%), drugs and medications (45.4%), and environmental exposures like smoking (37.7%). These gaps are concerning, as they may lead to under-recognition or delayed response to potential causes of HL. Though the general awareness is promising, tailored health education campaigns are essential to bridge the existing knowledge gaps. Increasing community knowledge about both the direct and indirect causes of HL, as well as the importance of early detection and intervention, could improve outcomes for the affected children. The study also explored a moderate level of Knowledge regarding preventive, diagnostic, and intervention measures. Participants recognized the benefits of exclusive breastfeeding for six months (61.6%) and routine childhood immunizations (57.1%) in lowering the risk of otitis media. Additionally, over half were aware of the possibility of early detection of HL shortly after birth (55.9%) and the availability of treatment options (59.4%). Participants also demonstrated a fair understanding of early signs of HL, with 71.1% recognizing speech and language delays as indicators. Furthermore, 82.3% believed that children with HL can be integrated into educational environments, indicating generally positive social attitudes.

Previous related studies conducted in Saudi Arabia and other countries have shown inconsistent results regarding both the general level of knowledge and the particular aspects where awareness of HL is either strong or lacking. A study conducted in the Qassim region of Saudi Arabia, involving 243 parents, reported a lower proportion of participants with a good level of knowledge (103, 42.4%). The highest awareness was observed for congenital HL, followed by head trauma. In contrast, the lowest levels of knowledge were related to jaundice and low birth weight (below 1500 g) [18]. Additionally, Alsuhaibani et al. [22] highlighted a lack of parental knowledge regarding otitis media in children and the preventive role of vaccination against acute otitis media. A more recent assessment of parental knowledge across the Kingdom of Saudi Arabia by Khurayzi et al. [23] reported that 58.4% of participating parents possessed a good level of understanding regarding childhood HL. However, the study also highlighted significant knowledge gaps, noting that more than half of both fathers and mothers were unaware that low birth weight, delayed crying at birth, and craniofacial anomalies are potential risk factors for HL.

Another study from the United Arab Emirates reported a lower percentage of parents with a good level of knowledge regarding the risk factors, diagnosis, and treatment of childhood HL (92 out of 269, 34.2%). While participants showed some awareness of congenital and genetic causes, there were notable gaps in knowledge about risk factors such as neonatal infections, jaundice, maternal rubella infection, certain medications, and the potential link between low birth weight and HL. Additionally, over half of the parents recognized that HL can be detected shortly after birth, is manageable, and that speech and language delays can be warning signs [21]. Furthermore, Altamimi and colleagues [24] reported a low level of awareness among Jordanian mothers regarding childhood HL. The study also emphasized several barriers, including limited access to reliable information, challenges in understanding medical advice, financial difficulties, and limited availability of healthcare services.

Interviews with mothers in Malaysia indicated a moderate level of knowledge about childhood HL, largely attributed to the absence of targeted community awareness programs for expectant mothers [25]. A more recent Malaysian study revealed that the majority of primary care physicians lacked adequate knowledge in this area, with 61.4% not considering childhood HL a significant health concern. Additionally, 45.9% were unaware that children with HL can succeed in mainstream schools. Awareness of risk factors among participants varied widely, ranging from 24.6% to 90.3% [26]. In contrast, a study conducted in Pakistan found that parents had an overall good level of knowledge (67.3%), with high awareness of congenital causes (79.3%) but low recognition of measles as a risk factor (29.3%) [27].

In India, Ravi et al. [28] found that mothers had relatively good awareness of prolonged noise exposure as a risk factor for HL (70.3%) but demonstrated limited knowledge about the role of drugs and medications in causing hearing impairment. Notably, 75% believed that treatment for HL is available and that affected children can attend school. Similarly, in Northern India, Sanju et al. [29] reported that 70% of the nurses surveyed were aware of the harmful effects of noise on infant hearing, but most lacked knowledge about the impact of hyperbilirubinemia on auditory health. The findings of another study conducted in Delhi involving 314 parents and caregivers indicated that parents had better knowledge than caregivers, with mothers showing higher awareness, particularly concerning noise exposure, medication use during pregnancy, and developmental communication milestones, while fathers were less engaged [30].

Furthermore, Kaspar and colleagues [20] evaluated parental awareness in the Solomon Islands and found high levels of knowledge regarding otitis media (94%), noise exposure (87.3%), and family history (72.7%) as risk factors for HL. However, there was limited awareness about jaundice and delayed crying at birth as potential causes. The study also reported strong parental understanding of the preventive role of routine childhood immunizations (84%) and breastfeeding (76%) in reducing the risk of otitis media.

The overall attitude toward childhood HL and related services was predominantly positive (73.1%). Most participating parents conveyed a strong willingness to engage with hearing health services for early detection and treatment of HL. They supported early testing (84.0%), school-based screening (87.3%), and hearing aid use (74.6%), though acceptance of ear surgery was somewhat lower (67.3%). Additionally, a majority of parents (88.5%) expressed a strong desire for more information regarding childhood HL. These findings agree with Kaspar et al. [20], who reported that 96% and 99.3% of parents accepted an infant hearing test and hearing tests at school, respectively. Additionally, both Alsudays et al. [18] and Khurayzi et al. [23] reported in their studies conducted in Saudi Arabia that the majority of parents held highly positive attitudes toward school-based hearing screening. A large proportion of fathers (79.4%) and mothers (74.3%) expressed strong support for having their children tested for HL in school settings. Studies from the United Arab Emirates [21], Jordan [24], Malaysia [26], and Pakistan [27] also reported similar positive attitudes.

The study also highlighted important sociodemographic influences on knowledge levels and attitudes. Older adults (aged ≥60) were significantly more likely to have limited knowledge, which may reflect generational differences in access to health information. In contrast, female participants tended to have better knowledge than male participants, which could be attributed to their traditionally more active roles in child healthcare and family health decisions. Participants with a medical background also demonstrated higher knowledge levels due to their familiarity with health-related issues. Alternatively, other factors, such as education level, did not show significant associations with awareness level. In this context, Kaspar et al. [20] observed that knowledge tended to improve with age, while Ayas and Yaseen [21] reported a significant association between age and level of knowledge, with greater knowledge levels among parents aged 31-40 years compared to other groups. Similar to our findings, Kaspar et al. [20], Ayas and Yaseen [21], and Alsudays et al. [18] found no association between knowledge and education level. However, Khurayzi et al. [23] observed a better level of knowledge among parents with high educational levels.

Concerning attitude toward hearing services, the findings indicate a notable gender and marital status disparity. Female participants were significantly more likely to exhibit a positive attitude, suggesting greater openness to the importance and utilization of hearing services. In contrast, male participants and widowed individuals significantly displayed negative attitudes. This could reflect differences in health-seeking behaviors, awareness, or social support systems. The more negative perception among widowed participants may also be related to psychosocial factors such as isolation or reduced access to information and resources. These findings dictate the need for targeted awareness and engagement strategies, particularly among male and widowed populations, to promote more favorable attitudes. Tailoring public health strategies to reach these subgroups can help ensure more reasonable awareness across the population [31].

This study has several strengths. The questionnaire was carefully designed based on previously validated instruments and relevant literature to ensure comprehensive coverage of parental knowledge and attitudes toward childhood hearing loss. It underwent rigorous content validation by domain experts and was translated into Arabic using a standardized forward-backward translation process to ensure linguistic and cultural accuracy. A pilot study was conducted to assess clarity and feasibility, and necessary modifications were made accordingly, enhancing the tool’s reliability and validity. These steps collectively strengthened the quality of the data obtained and the relevance of the findings to the Saudi context.

However, certain limitations should be acknowledged. First, being a cross-sectional survey, it captures information at a single point in time, thereby restricting the ability to establish causal relationships between variables. Second, the reliance on self-reported data introduces the possibility of response and recall bias, which may compromise the accuracy of the information provided by participants. Third, the use of convenience sampling could have resulted in selection bias, as individuals with a particular interest in the subject matter may have been more inclined to participate, potentially affecting the representativeness of the sample and the future generalization to the target population.

## Conclusions

This study provides important insights into parental knowledge and attitudes concerning childhood HL and hearing services in Tabuk City, Saudi Arabia. Findings revealed substantial variability in awareness of HL risk factors, with persistent gaps concerning less apparent causes such as neonatal jaundice, low birth weight, chemotherapy, and environmental exposures. While parents generally held positive views toward HL management and audiological care, motivation for certain interventions, particularly ear surgery, was more limited. Knowledge and attitudes were also shaped by sociodemographic characteristics, notably age and gender. These results underscore the need for targeted public-health strategies to broaden parental understanding of all risk factors for HL and to strengthen acceptance of evidence-based interventions.

## Appendices

Questionnaire

I. Participants’ Characteristics

Age (year) o 20 - 39 o 40 - 59 o ≥ 60 Gender o Female o Male Marital status o Married o Divorced o Widowed Number of children o 1 - 2 o ≥ 3 Level of education o Secondary school or below o University or above Monthly income (Saudi Riyal) o < 5,000 o 5,000 - 10,000 o > 10,000 Nationality o Saudi o Non-Saudi Medical background o Yes o No

II. *Participants’ Knowledge* 1. Babies can be born with hearing loss 2. Maternal infection can cause hearing loss 3. Neonatal infection can cause hearing loss 4. CNS infection can cause hearing loss 5. Drugs/medications can cause hearing loss 6. Radiotherapy can cause hearing loss 7. Chemotherapy can cause hearing loss 8. Jaundice can cause hearing loss 9. Family history can be associated with hearing loss 10. Consanguineous marriage can be associated with hearing loss 11. Low birth weight < 1500 g can be associated with hearing loss 12. Noise exposure can cause hearing loss 13. Head trauma can cause hearing loss 14. Otitis media and ear discharge can cause hearing loss 15. Breast-feeding for first 6 months can reduce/prevent otitis media 16. Smoking can predispose to otitis media 17. Routine childhood immunizations can reduce otitis media 18. Hearing loss can be identified soon after birth 19. Speech/language problems can be a sign of hearing loss 20. There is available treatment for hearing loss 21. Children with hearing loss can attend schools

III. *Participants’ Attitude* 1. I would like my baby tested soon after birth 2. I would like my child tested at school 3. I would let my child use hearing aids 4. I would accept ear surgery for my child 5. I would like more information
